# Hepatotoxicity with Vismodegib: An MD Anderson Cancer Center and Research on Adverse Drug Events and Reports Project

**DOI:** 10.1007/s40268-016-0168-2

**Published:** 2017-01-06

**Authors:** Beatrice J. Edwards, Dennis W. Raisch, Smita S. Saraykar, Ming Sun, Josh A. Hammel, Hai T. Tran, Nathaniel Wehr, Rasha Arabyat, Dennis P. West

**Affiliations:** 1 Department of General Internal Medicine, University of Texas MD Anderson Cancer Center, 1400 Pressler Dr., Unit # 1465, Houston, TX 77030 USA; 2 College of Pharmacy, University of New Mexico, Albuquerque, NM USA; 3 Department of Dermatology, Northwestern University, Chicago, IL USA; 4 Thoracic, Head and Neck Medical Oncology Research, University of Texas MD Anderson Cancer Center, Houston, TX USA; 5 Robert H. Lurie Comprehensive Cancer Center, Northwestern University, Chicago, IL USA

## Abstract

**Background:**

On 30 January 2012, the US FDA approved vismodegib (Erivedge^®^, Genentech, CA, USA) for the management of both metastatic and locally advanced basal cell carcinoma.

**Objective:**

Our objective was to identify evidence of hepatotoxicity with vismodegib in the FDA Adverse Event Reporting System (FAERS) in treated patients in two National Cancer Institute Comprehensive Cancer Centers.

**Methods:**

FAERS was searched for reports dated 1 January 2009 through 31 December 2015 using terms including hedgehog pathway and vismodegib and hepatic-related terms such as liver, jaundice, and hepatitis, among others. Disproportionality analyses with estimates of proportional reporting ratio and empirical Bayesian geometric mean were conducted. A comprehensive literature review was conducted, and the clinical databases at the University of Texas MD Anderson Cancer Center and Robert H. Lurie Comprehensive Cancer Center of Northwestern University were searched.

**Results:**

Two cases of severe liver dysfunction were published (Common Terminology Criteria for Adverse Events [CTCAE] class III), and 94 reports of adverse events (AEs) were detected in FAERS, 35 of which were serious AEs. Safety notifications related to hepatotoxicity have not been issued by the manufacturer or the FDA, although vismodegib is listed in LiverTox and the European Medicines Agency website.

**Conclusion:**

We identified a detectable safety signal for hepatotoxicity for vismodegib within 4 years of FDA approval. Vismodegib should be used in patients with severe liver disease only after careful consideration, and concomitant hepatotoxic medications should be avoided. Rapid dissemination of such safety concerns is expected to result in fewer serious hepatotoxic AEs and more optimal outcomes for patients with cancer receiving vismodegib.

**Electronic supplementary material:**

The online version of this article (doi:10.1007/s40268-016-0168-2) contains supplementary material, which is available to authorized users.

## Key Points

**Table:** 

Hepatotoxicity has been reported as an aderse event with Vismodegib.
Vismodegib is listed as a hepatotoxic medication in the LiverTox site. The European Medicine Agency reports the elevation of liver enzymes, and the Australian Therapeutic Goods Administration reported 3 cases of hepatotoxicty resulting in hospitalizatons with vismodegib.
The assessmen of liver profile prior to vismodegib use, and its avoidance in patients with moderate to severe liver disease is recommended. Consider avoidance of concomitant medications with hepatic metabolism. Recommend that safety recommendations be incorporated into the FDA website, and product package insert.

## Introduction

About eight in ten skin cancers are basal cell carcinomas (BCCs) [1], making it the most common type of skin cancer, arising from the lower layer of the skin. In 2014, more than 2 million estimated new cases and 1000 deaths occurred in the USA [2]. Surgery followed by radiation offers the most effective and efficient means for cure. Therapies such as 5-flurouracil, imiquimod, photodynamic therapy, or vigorous cryotherapy can be considered as alternative treatments for individuals with low-risk superficial BCC [3].

On 30 January 2012, the US FDA approved vismodegib for the treatment of recurrent, locally advanced, or metastatic BCC, based primarily on the results observed in a single-arm parallel-cohort international trial [4]. An overall response rate of 43 and 30% was seen in patients with localized and metastatic BCC [5].

The most common adverse events (AEs) associated with vismodegib reported in randomized controlled trials (RCTs) were muscle spasms, alopecia, dysgeusia, weight loss, fatigue, loss of appetite, and upper respiratory tract infections [4, 6]. One case of severe hepatotoxicity with vismodegib was reported in an 83-year-old female subject [7]. This individual’s medical history included hypertension, atrial fibrillation, and chronic obstructive pulmonary disease but no evidence of prior liver abnormalities. After 1 week of treatment, she developed nausea and emesis and was hospitalized. Liver function tests revealed a cholestatic pattern of hepatotoxicity, and liver biopsy exhibited nonspecific cholestasis with portal fibrosis. Other RCTs in advanced BCC [8] and ovarian cancer [9] have listed, less commonly, elevation of hepatic enzymes and abdominal pain.

The FDA Adverse Event Reporting System (FAERS) is a self-reported and voluntary database of AEs and medication errors reported to the FDA. It is designed to support the FDA’s post-marketing safety surveillance program for drugs and therapeutic biological products. FAERS data can be used to identify post-marketing safety signals. A safety signal is defined by the Working Group VIII of the Council for International Organizations of Medical Sciences (CIOMS VIII) as “Information that arises from one or multiple sources (including observations and experiments), which suggests a new potentially causal association, or a new aspect of a known association, between an intervention and an event or set of related events, that is judged to be of sufficient likelihood to justify further verification” [10]. Limitations include no proven causal relationship between events and a certain product and potential reporting bias [11].

The Research on Adverse Drug events And Reports (RADAR) project has received National Cancer Institute (NCI) and National Science Foundation (NSF) funding for pharmacovigilance activities. Based at Northwestern University, RADAR was first initiated in 1998 by a multidisciplinary investigator team that systematically investigated and disseminated information describing serious and previously unrecognized adverse drug and device reactions (ADRs) [12]. The purpose of this study was to determine whether a safety signal for vismodegib-related hepatotoxic AEs is detectable in FAERS and to search the clinical databases of two NCI-designated Comprehensive Cancer Centers in two large urban settings (Houston and Chicago, USA), The University of Texas MD Anderson Cancer Center and R.H. Lurie Comprehensive Cancer Center (RHLCCC) of Northwestern University, respectively.

## Methods

This retrospective study was approved by the Institutional Review Boards of MD Anderson Cancer Center and Northwestern University. RADAR methodology has been previously described in detail [12]. We searched FAERS for vismodegib and adverse liver event as combined terms from 1 January 2009 through 31 December 2015. All hepatic cases were identified through a search including ascites, hepatobiliary disease, liver toxicity, hepatitis, and cholestasis, among others. Complete search terms are listed in Appendix I in the Electronic Supplementary Material.

Within FAERS, we calculated disproportionate reporting of hepatic dysfunction using proportional reporting ratios (PRRs) with 95% confidence intervals (CIs). PRRs determined whether the proportion of FAERS cases with hepatic dysfunction was higher in patients receiving vismodegib than in those receiving other drugs. The PRR is a statistical aid that generates signals based on a proportionate approach, which also acknowledges the stability of a large database. PRRs involve calculation of the proportions of specified reactions for drugs of interest, with the comparator being the proportions of the specified reactions reported among all other drugs in the database. Judgment about the existence of a signal and its strength is made based on three pieces of information: the PRR, the chi-squared value of the data, and the number of cases. A signal is defined as a PRR of ≥2, a chi-squared value ≥4, and three or more cases reported to FAERS [12, 13].

The clinical databases at MD Anderson Cancer Center and RHLCCC were queried to detect individuals exposed to vismodegib. Medical records of these cases were reviewed, and data on demographics (age, sex, race/ethnicity, type of cancer) and liver function tests were collected. In addition, three major electronic databases were searched (MEDLINE, PubMed, and Embase) for peer-reviewed articles published between 1970 and 31 March 2014 using terms such as vismodegib, GDC-0449, sonic hedgehog, neoplasm, and clinical trials. The literature review obtained information on the results of clinical trials of vismodegib reporting on efficacy and adverse effects. We also searched websites and safety databases maintained by the FDA and other major international regulatory agencies.

Common Terminology Criteria for Adverse Events (CTCAE) version 4.0 was used as toxicity grading criteria for AE reporting. A grading (severity) scale is provided for each AE term (grade 1: mild AE; grade 2: moderate AE; grade 3: severe AE; grade 4: life-threatening or disabling AE; grade 5: death related to AE) [14]. For hepatotoxicity cases detected within FAERS, concomitant drugs were reviewed; those with significant hepatic metabolism were identified based on information contained within multiple databases [15].

## Results

### FDA Adverse Event Reporting System (FAERS) Results

In total, 94 FAERS cases were reported as having at least one AE of liver dysfunction, with 35 reports including terms demonstrating severe hepatotoxicity (Table 1), all of which were reported to FAERS by the sponsor. Of 35 reports, 20 were serious AEs (SAEs) resulting in hospitalization or death. Other AEs with these cases included dysphagia, abdominal discomfort, and cognitive disorders. The mean age of all 35 individuals was 60 years (range 0–98); 16 were female and 16 were male (of the 32 FAERS cases in which sex was reported). Comorbidities were infrequently reported and included hypertension (3/35), diabetes mellitus (2/35), hypothyroidism (1/35), and acute respiratory distress syndrome (1/35). Signal-detection analyses resulted in PRRs of 2.71 (95% CI 2.3–3.2) among all hepatic dysfunction cases and 2.24 (95% CI 1.7–2.9) among serious hepatotoxicity cases, and reflect a significant safety signal.

**Table 1:** Cases of hepatotoxicity attributed to vismodegib in the FDA Adverse Event Reporting System

Report date	Age^a^ (sex)	Cancer type	Concomitant drugs with hepatic metabolism	Co-morbidities	Adverse events	CTCAE	Outcomes	Vismodegib administration time course (days)
2012	83 (F)	BCC	Lanoxin, Lasix, Theophylline	NA	Hepatocellular injury	4	Hospitalization, life threatening	NA
2012	84 (M)	BCC	NA	NA	Hepatitis	4	Life threatening	47
2012	63 (F)	BCC	Cymbalta, Levothyroxine	NA	Hepatotoxicity	3	Hospitalization	55
2012	58 (NA)	Pancreatic carcinoma metastatic	Oxycontin	NA	Ascites	5	Death, hospitalization	218
2012	59 (M)	Adenocarcinoma pancreas	NA	NA	Ascites	3	Hospitalization	5
2012	41 (F)	BCC	NA	NA	Hepatitis	3	Hospitalization	60
2013	NA (F)	BCC	NA	NA	DILI	NA	Other	NA
2013	50 (M)	BCC	NA	NA	Hepatitis	4	Hospitalization, life threatening	NA
2013	76 (M)	BCC	Uroxatral, Zocor, Zyloprim	NA	Hepatotoxicity	4	Hospitalization	NA
2013	59 (F)	BCC	Advair, Premarin	Asthma	Hepatitis	3	Hospitalization	NA
2013	68 (F)	NA	Diovan, Vibramycin	NA	DILI	NA	Other	NA
2013	NA	NA	NA	NA	Liver disorder	NA	Other	NA
2014	75 (M)	BCC	NA	NA	Hepatocellular injury	4	Disability other	52
2014	79 (F)	AML	Augmentin, Diovan, Dulcolax, Hydrea, Hydrochlorothiazid, Lipitor, Nystatin, Tenormin, Zyloprim	Autoimmune thyroiditis, hyperlipidemia, hypertension	Hepatic infection	3	Hospitalization	NA
2014	98 (F)	BCC	NA	NA	DILI	NA	Other	48
2014	90 (M)	BCC	Amoxicillin, Aspirin, Kerlone, Lotensin, Lozol, Norvasc, Rutinoscorbin, Tricor, Ultracet, Vinpocetine, Zocor	Hypercholesterolemia, hyperlipidemia, hypertension	DILI	3	Hospitalization	66
2014	72 (M)	BCC	Cialis, Diovan, Norvasc		Cholestatic liver injury	3	Hospitalization	NA
2014	68 (M)	BCC	Amaryl, Bactrim, Capoten, Cardizem, Coumadin, Glucophage, Jentadueto, Lantus, Pravachol, Ranexa, Xarelto	Hyperlipidemia, hypertension, T2DM	Acute hepatic failure	5	Hospitalization, disability, death	217
2014	88 (M)	BCC	NA	NA	Hepatitis cholestatic	3	Hospitalization	NA
2015	70 (M)	NA	NA	NA	Hepatotoxicity	NA	Other	NA
2015	72 (M)	BCC	NA	NA	Liver injury	NA	Other	NA
2015	48 (F)	NA	NA	NA	Hepatic failure	NA	Other	NA
2015	0 (F)	NA	NA	NA	Liver disorder	NA	Other	NA
2015	56 (M)	NA	NA	NA	Hepatobiliary disease	5	Hospitalization, death	18
2015	70 (F)	BCC	Diosmectite, Elavil, Hexaquine, Miralax, Nexium, Tylenol, Ultracet, Ultram, Vitabact	DM	Ascites	3	Hospitalization	853
2015	68 (M)	NA	Capoten, Cardizem, Colace, Diabeta, Eliquis, Glucophage, Jentadueto, Lantus, Lasix, Lopressor, Nitroglycerin, Plavix, Pravachol, Protonix, Ranexa, Sulfamethoxazole	NA	Acute hepatic failure	5	Hospitalization, life threatening, death	240
2015	83 (F)	BCC	NA	NA	Hepatocellular injury	NA	Other	81
2015	0 (F)	NA	NA	NA	Hepatotoxicity	NA	Other	NA
2015	0 (M)	NA	Diovan, Fusidic Acid, Glucophage, Januvia, Lipitor, Robaxacet, Sectral, Symbicort, Ventolin, Veramyst, Voltaren	NA	Hepatitis	3	Hospitalization	NA
2015	76 (F)	NA	Enbrel, Hyzaar	NA	Hepatitis toxic	3	Hospitalization	21
2015	89 (F)	NA	NA	NA	Hepatitis	NA	Other	NA
2015	0 (NA)	NA	NA	NA	Liver disorder	3	Hospitalization	NA
2015	71 (F)	BCC	NA	NA	Hepatitis acute	NA	Other	NA
2015	57 (M)	BCC	NA	NA	Hepatic failure	5	Death	NA
2015	0 (M)	BCC	NA	NA	Hepatitis cholestatic	NA	Other	NA

Assessment based on FAERS summary data. Since FAERS case data are often incomplete, missing data are shown as ‘NA’

*AML* acute myeloid leukemia, *BCC* basal cell carcinoma, *CTCAE* Common Terminology Criteria for Adverse Events, Version 4.0, *DILI* drug-induced liver injury, *DM* diabetes mellitus, *F* female, *M* male, *NA* not available, *T2DM* type 2 diabetes mellitus

^a^Age is presented in years

### University of Texas MD Anderson Cancer Center and Robert H. Lurie Cancer Center Results

In total, 15 patients received vismodegib in two cancer centers: for BCC (*n* = 13) and for medulloblastoma (*n* = 2). Two cases experienced elevated alkaline phosphatase (twofold elevation).

## Discussion

**Fig. 1:**
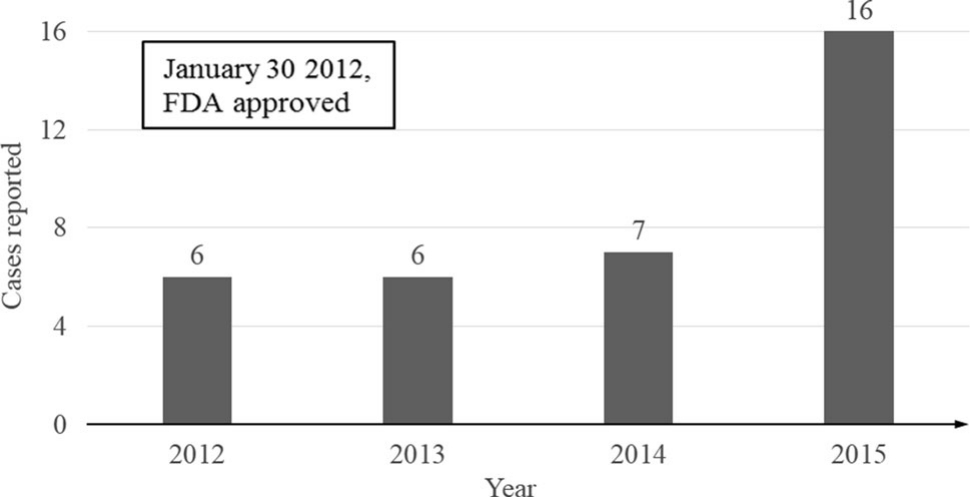
Severe cases reported in the US FDA Adverse Event Reporting System by year

We ascertained that hepatic events associated with vismodegib in FAERS produced a safety signal. This association was previously described in the study by Ventarola and Silverstein [16]. We identified an increasing incidence over the extended study period. The first case was reported prior to FDA approval in 2011, and there has been an upward trend in cases reported to FAERs over the last 4 years (Fig. 1). It has been reported that a median of 7 years post-marketing elapses before newly detected SAEs are announced by pharmaceutical suppliers and/or the FDA [17], but this report of vismodegib-associated hepatotoxicity is being disseminated within 4 years of approval. The importance of systematic interrogation and analyses with large databases such as FAERS is made evident in that, in two NCI-designated comprehensive cancer centers, initial interrogation showed no detectable reports of hepatotoxicity. Such a finding is not unexpected, given the small number of treated patients (*n* = 15) in two of the NCI-designated 41 comprehensive cancer centers. Since hepatotoxicity may progress to be severe and/or life threatening, further exploration of this important association is essential. According to the NCI, only about 15% of US cancer patients are diagnosed and treated at the nation’s major academic-based cancer centers, and the large remainder may not be easily tracked for outcomes [18]. Moreover, since FAERS case reports are de-identified, it is not practical to ascertain, and/or contact the initial reporter for, further clinical information and outcomes. Moreover, FAERS reports of vismodegib-associated hepatotoxicity typically do not provide detail on related comorbidities such as prior liver abnormalities or alcohol abuse.

Some possible mechanisms of drug-related hepatotoxicity include disruption of the cell membrane and drug binding to cell protein, leading to cell death; cell apoptosis; inhibition of cell mitochondria function; cellular pathway inhibition; and abnormal bile flow leading to cholestasis and jaundice. Moreover, obesity and malnutrition, pregnancy, history of drug reactions, and pre-existing liver disease are factors that lead to susceptibility to hepatotoxicity [19]. Liver abnormalities may also include etiological factors such as nonalcoholic steatohepatitis (NASH), medication use, alcohol use, and chronic liver disease, among others [20].

In published RCTs, vismodegib resulted in elevated alanine aminotransferase levels compared with the placebo arm [21]. SAEs (CTCAE grade 3–4) were seen in two participants in an ovarian cancer RCT [9]. Hedgehog signaling has generally been considered inactive in healthy adult hepatocytes. Paracrine connections with hepatic epithelial cells are important for embryonic development, cell proliferation, and recovery from chronic liver damage [22–24]. Vismodegib functions as a small-molecule inhibitor of the hedgehog pathway by binding to smoothened (SMO) receptors in cells. Therefore, it may be reasonable to hypothesize that the damage caused by NASH creates an idiosyncratic trait within patients, placing them at a higher risk for experiencing a liver-based AE if they are treated with vismodegib as the drug attempts to regulate a highly damaged system.

Concomitant medication use, summarized in FAERS for 14 of the serious cases, may prove to be quite important, as four of the reports in FAERS indicated concomitant use of acetaminophen, a common over-the-counter medication (Table 1). Acetaminophen may lead to acute hepatotoxicity or necrosis, especially with overdose; acetaminophen poisoning accounts for nearly 50% of all cases of acute liver failure in the USA [25]. Ash and Jolly [26] reported a case of acute liver injury related to a possible drug–drug interaction between vismodegib, aspirin, and naproxen. Thus, concomitant drug use with vismodegib may contribute to drug–drug interactions that may play a major role in the development of liver toxicity.

The press announcement at the time of the original FDA approval on 30 January 2012 and the DailyMed website, on which full prescribing information documents are posted by the US National Library of Medicine, both specified that safety and effectiveness were not established for patients with hepatic impairment who receive vismodegib [27–29]. LiverTox currently lists vismodegib as a potentially hepatotoxic medication [30]. Health Canada has a statement similar to that of the FDA in relation to liver impairment but with an arrangement whereby the sponsor agrees to submit additional safety studies, specifically to include results from a renal/hepatic impairment study by March 2015 [31]. The European Medicines Agency (EMA) reported that data on moderate and severe liver impairment were too limited to enable conclusions about liver impairment, but nevertheless posted a table including abnormal liver enzymes as a common side effect in 1–10% of patients [32]. The Australian Government’s Therapeutic Goods Administration released a statement citing study SHH4489g, which attributed three cases of hepatotoxicity to vismodegib, cases resulted in hospitalizations, but no deaths were reported [33]. The agency recommended vismodegib be avoided in patients with severe hepatic impairment [33]. As such, although the FDA labeling (full prescribing information) mentions that medications that are liver metabolized should be avoided, it provides no information on drug interaction risks for vismodegib [27, 28]. Especially in light of healthcare system reform, busy health practitioners must access and utilize ever-increasing amounts of information in the prescribing of medications and patient management, with a continuous need for new and updated post-marketing risk information and newly identified clinically significant drug interactions [34].

In contrast with other drug regulatory agencies, the FDA has not yet advanced newly evolving risk information regarding vismodegib and hepatotoxicity, although it has issued recommendations for studies of vismodegib use in individuals with renal and liver dysfunction [29]. Patients with mild liver impairment should be carefully monitored if vismodegib is considered for therapy. We also recommend that potentially hepatotoxic agents such as acetaminophen and alcohol be avoided during vismodegib therapy.

Limits of this study may include under-reporting and potential reporting bias because of the voluntary nature of the FAERS database and the impracticability of contacting AE reporters. As such, the ability to establish causality of hepatotoxicity associated with vismodegib based on FAERS data is limited, albeit significant signal detection values were found.

## Conclusion

Given a detectable safety signal for hepatotoxicity within FAERS for vismodegib, its use in patients with severe liver disease should include careful consideration of risk versus benefit. We recommend close monitoring of liver function and cautious use with concomitant hepatotoxic medications. As such, the FDA decision to waive Risk Evaluation and Mitigation Strategies (REMS) as part of its vismodegib labeling may require re-evaluation and carefully conducted prospective pharmacovigilance studies to achieve safe use. Rapid dissemination of such safety concerns is expected to result in fewer serious hepatotoxic adverse events and more optimal outcomes for patients with cancer receiving vismodegib.

## Electronic supplementary material

Below is the link to the electronic supplementary material.

Supplementary material 1 (DOCX 13 kb)
