# Supplementary material for: Hepatotoxicity with Vismodegib: An MD Anderson Cancer Center and Research on Adverse Drug Events and Reports Project
Source: Drugs R D. 2017 Jan 6;17(1):211–8. doi: 10.1007/s40268-016-0168-2 (PMC5318336; doi:10.1007/s40268-016-0168-2)
Supplement: Supplementary file 1 — Supplementary material 1 (DOCX 13 kb) [file 40268_2016_168_MOESM1_ESM.docx]

Appendix I. search terms used in FAERS

All hepatic cases

| MedDRA PT |
| --- |
| Ascites |
| Hepatobiliary disease |
| Cholangitis acute |
| Hepatitis toxic |
| Drug-induced liver injury |
| Hepatotoxicity |
| Cholestasis |
| Hepatitis cholestatic |
| Hepatitis acute |
| Hepatocellular injury |
| Hepatitis |
| Cholecystitis acute |
| Acute hepatic failure |
| Liver injury |
| Cholelithiasis |
| Cholecystitis |
| Liver disorder |
| Hepatic failure |
| Hepatic function abnormal |
| Blood bilirubin decreased |
| Blood bilirubin abnormal |
| Aspartate aminotransferase decreased |
| Hepatic enzyme increased |
| Hyperbilirubinaemia |
| Transaminases increased |
| Blood alkaline phosphatase increased |
| Gamma-glutamyltransferase increased |
| Blood bilirubin increased |
| Jaundice |
| Aspartate aminotransferase increased |
| Liver function test abnormal |
| Alanine aminotransferase increased |

All SERIOUS hepatic terms

| MedDRA PT |
| --- |
| Ascites |
| Hepatobiliary disease |
| Cholangitis acute |
| Hepatitis toxic |
| Drug-induced liver injury |
| Hepatotoxicity |
| Cholestasis |
| Hepatitis cholestatic |
| Hepatitis acute |
| Hepatocellular injury |
| Hepatitis |
| Cholecystitis acute |
| Acute hepatic failure |
| Liver injury |
| Cholelithiasis |
| Cholecystitis |
| Liver disorder |
| Hepatic failure |
| Hepatic function abnormal |
